# Prospective phase II trial of pazopanib plus CapeOX (capecitabine and oxaliplatin) in previously untreated patients with advanced gastric cancer

**DOI:** 10.18632/oncotarget.8175

**Published:** 2016-03-18

**Authors:** Seung Tae Kim, Jeeyun Lee, Su Jin Lee, Se Hoon Park, Sin-Ho Jung, Young Suk Park, Ho Yeong Lim, Won Ki Kang, Joon Oh Park

**Affiliations:** ^1^ Division of Hematology-Oncology, Department of Medicine, Samsung Medical Center, Sungkyunkwan University School of Medicine, Seoul, Korea; ^2^ Center of Biostatistics and Clinical Epidemiology, Samsung Medical Center, Sungkyunkwan University School of Medicine, Seoul, Korea

**Keywords:** pazopanib, capecitabine, oxaliplatin, gastric cancer

## Abstract

We designed a single-arm, open label phase II study to determine the efficacy and toxicity of the combination of pazopanib with CapeOx (capecitabine and oxaliplatin) in metastatic /recurrent advanced gastric cancer (AGC) patients. Previously untreated AGC patients received capecitabine (850 mg/m^2^ bid, day 1–14) plus oxaliplatin (130 mg/m^2^, day 1) in combination with pazopanib (800 mg, day 1–21) every three weeks. Treatment was continued until progression of the disease or intolerable toxicity was observed. In all, 66 patients were treated with pazopanib plus CapeOx. The median age of the patients was 51.5 years (range, 23.0–77), and the median ECOG performance status was 1 (0–1). Among all 66 patients, one complete response and 37 partial responses were observed (overall response rate, 62.4%; 95% confidence interval (CI), 45.7–73.5% accounting for the 2-stage design of this trial). Stable disease was observed in 23 patients (34.8%), revealing a 92.4% disease control rate. The median progression free survival and overall survival were 6.5 months (95% CI, 5.6–7.4) and 10.5 months (95% CI, 8.1–12.9), respectively. Thirty-four patients (51.5%) experienced a treatment-related toxicity of grade 3 or more. The most common toxicities of grade 3 or more were neutropenia (15.1%), anemia (10.6%), thrombocytopenia (10.6%), anorexia (7.6%), nausea (3.0%), and vomiting (3.0%). There were no treatment-related deaths. The combination of pazopanib and CapeOx showed moderate activity and an acceptable toxicity profile as a first-line treatment in metastatic / recurrent AGC patients (ClinicalTrials.gov NCT01130805).

## INTRODUCTION

Gastric cancer (GC) is the second most common cause of cancer-related death worldwide and the most frequently occurring malignancy in Korea [1, 2]. Although most patients with early stage disease receive surgical resection with curative intent, more than 60% of these patients have a high rate of locoregional as well as distant recurrence [3–5]. For patients with unresectable, recurrent, or advanced gastric cancer (AGC), systemic chemotherapy can improve survival and symptom control. Combination chemotherapy improves treatment outcomes compared with mono-chemotherapy or best supportive care in patients with advanced gastric cancer [6]. Although there is no internationally accepted standard first-line chemotherapy regimen, either infusional or oral fluoropyrimidine plus a platinum compound is now regarded as a standard regimen. However, more than half of the patients with AGC who receive standard chemotherapy do not achieve a response, and even in responders, the duration of their response was as short as a few months [7, 8]. Moreover, the role of molecularly targeted therapy has not been adequately explored in AGC when compared with other common solid tumors, such as non-small cell lung cancer, breast, and colorectal cancer.

The vascular endothelial growth factor (VEGF) pathway is involved in angiogenesis and is a commonly targeted pathway in oncology in order to decrease the tumor's vascular supply and metastasis, leading to tumor shrinkage. VEGF receptor (VEGFR) types 1 and 2 are the two receptors that are primarily responsible for mediating the angiogenic signals. In addition to directly inhibiting tumor-associated angiogenesis, which is necessary for tumor growth, antiangiogenic therapy may normalize “leaky” tumor vasculature and improve the availability of cytotoxics at the tumor site, consequently improving the clinical benefit [9]. Previous studies have shown that combining antiangiogenic agents with conventional cytotoxic chemotherapy encourages antitumor activity and improves toxicity profiles [10–12].

Pazopanib (GW786034; GlaxoSmithKline, Stevenage, UK) is a novel oral multitargeted tyrosine kinase inhibitor with a wide range of activities that are mediated through the VEGF receptor (VEGFR) types 1, 2, and 3, platelet-derived growth factor receptors α and β, and stem cell factor receptor (c-kit) [13, 14]. The anti-tumor efficacy of pazopanib has been demonstrated against a broad range of human tumors in both preclinical models and clinical studies [15, 16]. Pazopanib has been approved for the treatment of renal cell carcinoma (RCC) and soft tissue sarcoma based on large randomized phase III trials [17, 18]. In addition to anti-tumor activity, pazopanib is known to have a more tolerable toxicity profile and patients treated with this drug have a more favorable health-related quality of life (HRQoL) than patients with some other agents [19]. These advantages are important factors to consider in a palliative setting. Various clinical trials incorporated pazopanib in combination with reference cytotoxic regimens of various solid cancers [20–22]. CapeOx (capecitabine and oxaliplatin), the reference regimen of AGC and colorectal cancer (CRC), was studied in combination with pazopanib. In a phase I pazopanib plus CapeOx trial with 29 CRC patients, pazopanib (800 mg) plus modified CapeOx (capecitabine 850 mg/m^2^ and oxaliplatin 130 mg/m^2^) were considered to be the optimally tolerated regimen [23]. A response rate of 38% was reported in CRC. Considering the clinical evidence of its efficacy and the favorable toxicity profile, the addition of pazopanib to CapeOx might be a reasonable candidate for palliative chemotherapy in AGC.

We designed a single-arm, open label phase II study to determine the efficacy and toxicity of the combination of pazopanib with CapeOx in metastatic and/or recurrent AGC patients.

## RESULTS

### Patient characteristics

The clinicopathologic characteristics of the 66 patients enrolled in this study are summarized in Table 1. The median age of the patients was 51.5 years (range, 23–77 years) and the majority of the patients were male (57.6%). The median ECOG performance status was 1 (0–1), and 24 patients had recurrent disease at study entry. All 66 patients had adenocarcinoma, 75.8% of whom had poorly differentiated or signet-ring cell-type disease. The major involved organs were the intra-abdominal lymph nodes and peritoneum.

**Table 1:** Baseline characteristics of the patients in this study (*N* = 66)

Characteristics	No. of patients	%
Age		
Median (Range)	51.5 (23.0–77.0)	
ECOG performance status		
Median (Range)	1 (0–1)	
Gender		
Male	38	57.6
Female	28	42.4
Disease status		
Recurrent	24	36.4
Metastatic	42	63.6
Pathologic type		
Well or moderately differentiated	16	24.2
Poorly differentiated or signet ring	50	75.8
Metastatic site		
Lymph node	41	62.1
Liver	12	18.2
Lung	4	6.1
Peritoneum	29	43.9
Bone	8	12.1
No. of metastatic lesions		
1	35	53.0
2	21	31.8
3 ≤	10	15.2

*ECOG* Eastern Cooperative Oncology Group.

### Delivery of drugs

The median number of treatments was 6.0 cycles (range, 1–20 cycles). Twenty-seven patients (40.9%) received eight or more cycles of treatment. The average relative dose-intensities were 0.87 for pazopanib, 0.78 for capecitabine, and 0.89 for oxaliplatin. Of the 66 patients, 40 (60.6%) had their pazopanib dose held or modified, per protocol, at some point during the study.

### Efficacy

**Figure 1:**
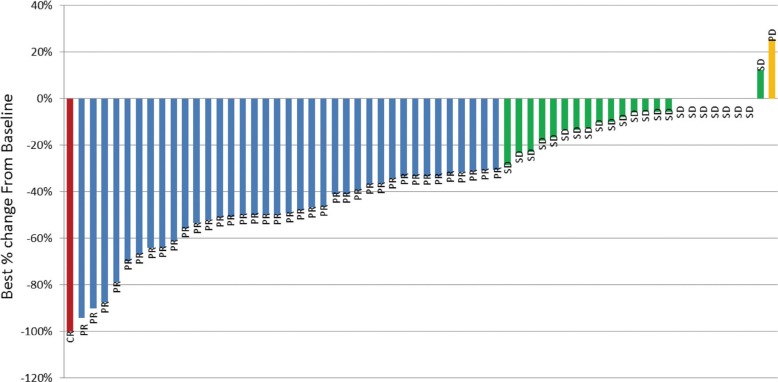
Maximum best change in tumor size from baseline Decreased in best percent change from baseline = 100%. Red bar complete response, Blue bar partial response, Green bar stable disease, Yellow bar progressive disease.

In the 66 eligible and treated patients, one complete response and 37 partial responses were observed (overall RR, 62.4%; 95% confidence interval (CI), 45.7 – 73.5%). For testing the hypothesis that the true overall RR is higher than 50%, the *p*-value was estimated to be 0.0993. Since this is smaller than alpha = 0.1, so that we accepted the study therapy for further investigation. The maximum best change observed was a 100% decrease in sum of longest diameters when compared with baseline (Figure 1). An additional 23 patients (34.8%) achieved stabilization of their disease (Table 2). Only one patient had confirmed disease progression at their first disease assessment. Forty-one patients (62.1%) acquired early tumor shrinkage (ETS) that was defined as a ≥ 10% decrease of the sum of the longest diameter of the target lesions six weeks after treatment. There were four patients whose disease status was not evaluable for treatment response because they were lost to follow-up.

**Table 2:** Treatment response of enrolled patients

Response	No. of patients	%
Complete response	1	1.5
Partial response	37	56.1
Stable disease	23	34.8
Progressive disease	1	3.0
Not available	4	6.1
Overall response rate	38	57.6
Disease control rate	61	92.4
Early tumor response at six weeks	41	62.1

**Figure 2:**
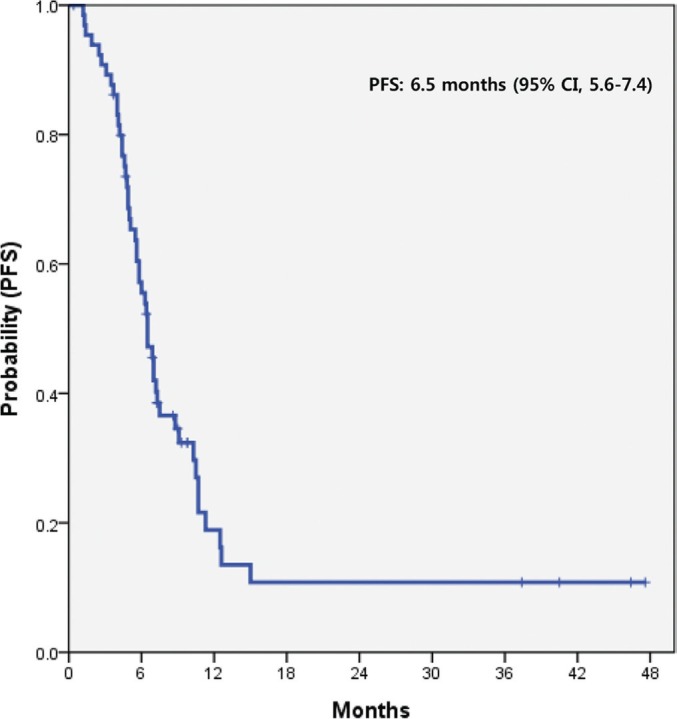
PFS of AGC patients treated with pazopanib and CapeOx *PFS* progression-free survival, *AGC* advanced gastric cancer, *CI* confidence interval, *CapeOx* capecitabine and oxaliplatin.

**Figure 3:**
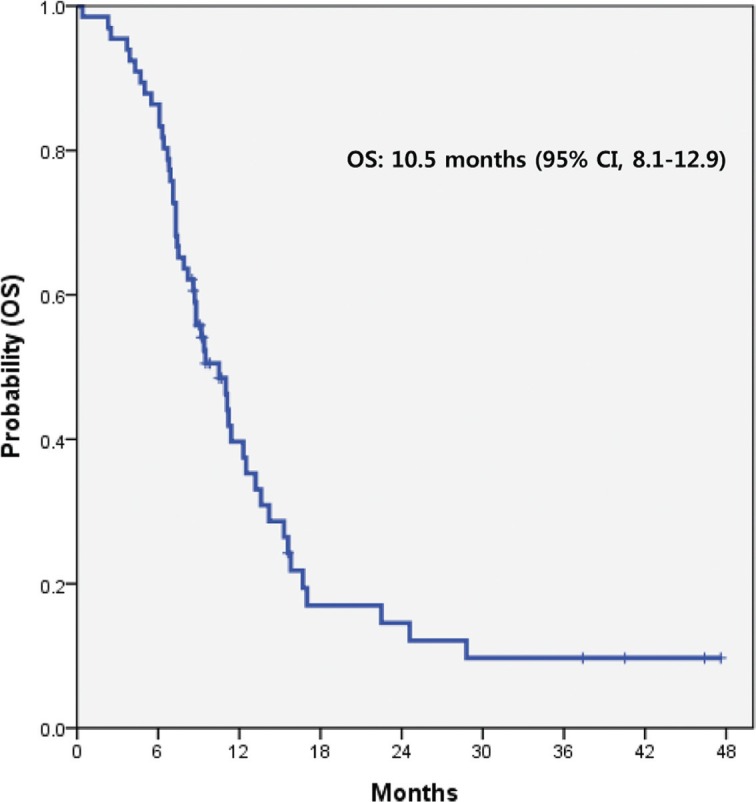
OS of AGC patients treated with pazopanib and CapeOx *OS* overall survival, *AGC* advanced gastric cancer, *CI* confidence interval, *CapeOx* capecitabine and oxaliplatin.

All 66 patients were included in the survival analysis with an intent-to-treat basis. At the time of data analysis, 54 (81.8%) of the 66 enrolled and treated patients had experienced disease progression and 50 (75.7%) patients were known to have died. The median PFS was 6.5 months (95% CI, 5.6–7.4 months) (Figure 2). The median OS was 10.5 months (95% CI, 8.1–12.9 months) (Figure 3). There was no significant difference in PFS and OS between patients with and without ETS (*p* > 0.05).

### Toxicity

The safety population included patients who were treated with at least one dose of the study medication. All 66 patients were assessable for toxicities. Thirty-four patients experienced a treatment-related toxicity of grade 3 or more during the study (Table 3). The most common toxicities of grade 3 and 4 were neutropenia (15.1% of all patients), anemia (10.6%), thrombocytopenia (10.6%), anorexia (7.6%), nausea (3.0%), and vomiting (3.0%). ALT/AST elevation and electrolyte disturbance (hypokalemia) were each reported in one patient. There was no case with hand foot syndrome of grade 3 or more. However, hand foot syndrome of grade 1 or 2 occurred in 32% of patients. There were no treatment-related deaths.

**Table 3:** Grade 3/4 Adverse Events (*N* = 66)

Toxicity	Number of patients (%) (*N* = 34)
Anemia	7 (10.6)
Neutropenia	10 (15.1)
Thrombocytopenia	7 (10.6)
Anorexia	5 (7.6)
Nausea	2 (3.0)
Vomiting	2 (3.0)
Diarrhea	1 (1.5)
Neuropathy	1 (1.5)
ALT elevation	1 (1.5)
AST elevation	1 (1.5)
Hypokalemia	1 (1.5)

*ALT* alanine transaminase, *AST* aspartate aminotransferase.

## DISCUSSION

The improvement in the treatment of AGC has plateaued although newer chemotherapeutic agents have been introduced [7, 8, 24]. Recently, advances in molecular biology have induced the development of many molecularly targeted agents. Currently, to improve the treatment-outcome, various clinical trials have tried to incorporate novel, molecularly targeted agents in combination chemotherapy for AGC [25–28]. This study is the first trial that evaluated the effect of incorporating pazopanib with CapeOx (capecitabine and oxaliplatin) in previously untreated metastatic and/or recurrent AGC. The response rate of pazopanib plus CapeOX was 62.4% (95% CI, 45.7–73.5%) and toxicity-profiles were acceptable. Therefore, these outcomes support the necessity of further definite trials for the incorporation of pazopanib with chemotherapy in AGC.

RRs as a frontline of various combination chemotherapy regimens ranged from 30% to 70% [8, 29–31]. Recently, adding novel molecular targeted agents to combination chemotherapy has been increasingly tested as a first-line treatment in AGC. The combination of sunitinib with capecitabine and cisplatin or oxaliplatin revealed RRs of 46.7% and 43.5%, respectively [32]. The RR for sorafenib, docetaxel, and cisplatin was reported to be 41% [25]. In this study, the combination of pazopanib and CapeOx showed a RR of 57.6%. This RR compares favorably to that of various previously reported novel, targeted agents plus chemotherapy regimens. In our study, the PFS was 6.5 months. The PFS of our regimen also compares favorably to the PFS of 5.8 months obtained with sorafenib, docetaxel, and cisplatin. These findings suggest that pazopanib may contribute additional anti-tumor effects to the CapeOx in previously untreated metastatic and/or recurrent AGC.

Our regimen showed an acceptable and manageable toxicity profile. Thirty-four patients (51.5%) experienced a treatment-related toxicity of grade 3 or more during the study. There were no extra or unexpected toxicities. The strategy of adding molecular-targeted agents to chemotherapy may be of concern in terms of the possibility for increasing toxicities. Generally, patients with recurrent or metastatic AGC are receiving palliative treatment. For these patients, the tolerability and toxicity of treatments are considered to be as important as the efficacy of the treatment. According to trials for combined sorafenib or bevacizumab with chemotherapy in the same setting, 91% and 84% of patients experienced grade 3 or more toxicities, respectively [25, 33]. Pazopanib has been known to have favorable toxicity profiles. In a clinical trial for metastatic RCC, pazopanib showed similar efficacy as a standard targeted agent [17]. However, the safety and QoL profiles favored pazopanib. Another study also demonstrated a significant patient preference for pazopanib over other standard targeted agent due to less fatigue and better overall QoL [19]. These finding suggested that pazopanib might be a good candidate targeted agent that could be incorporated with combination chemotherapy. Although this study did not analyze the QoL of patients or directly compare pazopanib to other targeted agents, this regimen (pazopanib plus CapeOx) appeared to have a lower frequency of grade 3 or higher toxicities than other regimens combining targeted agents as a counterpart of combination chemotherapy.

Antiangiogenic therapies are known to decrease tumor vascularization rather than result in direct cytotoxicity, and they have been associated with reduced tumor shrinkage compared to traditional antitumor agents [34–36]. In metastatic RCC, the 10% tumor shrinkage at first follow-up after anti-VEGF targeted agents, such as sunitinib, sorafenib or bevacizumab, has been known as the best predictor of survival [37, 38]. We evaluated the role of the tumor shrinkage at first follow-up in AGC patients receiving pazopanib containing combination therapy. We used a cutoff value of a 10% decrease in tumor size at six weeks as the criterion for ETS. This value was previously used as a cutoff to predict an improved outcome in Choi's criteria for gastrointestinal stromal tumors treated with imatinib and metastatic colorectal cancer treated with cetuximab [39–41]. The significance of this apparently rather small decrease might be related to the number of cancer cells actually eradicated by treatment; in a spherical tumor, 10% shrinkage would indicate that almost 30% of cells have been killed [42]. In this study, 10% tumor shrinkage was not a reliable early predictor of outcome. This discordance may be caused by the difference in the degree and the importance of vascularity between RCC and AGC, the use of combined cytotoxic chemotherapies, and the difference in the effect between pazopanib and anti-VEGF targeted agents.

This study had some drawbacks as a single-arm phase II trial, such as the limited sample size, patient selection, heterogeneous disease, and possible enrollment bias. In this study, 21 patients received the combination with capecitabine plus pazopanib after CapeOx of 8 cycles. Until now, whether the continuation of treatment including maintenance strategy in metastatic/relapsed gastric cancer is benefit is not clear. Oyan et al. reported that capecitabine maintenance might be promising in advanced gastric cancer. However, to confirming the efficacy and safety of CapeOx with capecitabine-maintenance in AGC, further clinical trials are needed. Nevertheless, this regimen (pazopanib plus CapeOx) showed moderate activity and an acceptable toxicity profile as a first-line treatment in metastatic and/or recurrent AGC patients. Pazopanib may contribute additional anti-tumor effects to chemotherapy while maintaining appropriate tolerability. Further investigation of pazopanib in combination with chemotherapy for AGC is worth conducting.

## PATIENTS AND METHODS

### Eligibility

Patients enrolled in this study had measurable, histologically confirmed metastatic and/or recurrent gastric adenocarcinoma. Baseline imaging work-ups were conducted within four weeks of entry into the study. They were required to be at least 18 years old and have at least one measurable lesion and an Eastern Cooperative Oncology Group (ECOG) performance status of 0 or 1. Previous adjuvant treatment, such as chemotherapy or chemo-radiotherapy, was allowed. Any other radiotherapy, chemotherapy or investigational therapies were not permitted. Adequate hematologic function (absolute neutrophil count ≥ 1.5 × 10^9^/L, platelet count ≥ 100 × 10^9^/L), hepatic function (aspartate aminotransferase/alanine aminotransferase (AST/ALT) ≤ 2.5 times the upper normal limit (UNL), total bilirubin b1.5 times the UNL), and renal function (serum creatinine ≤ 1.5 times the UNL) were required. Patients were required to not have an acute active infection. A prior history of another malignancy within five years of entry into the study, apart from nonmelanoma skin cancer or carcinoma *in situ* of the uterine cervix, precluded participation in this study. Women could not be pregnant or breast-feeding, and women with childbearing potential and sexually active males were strongly advised to use an effective method of contraception. Patients with known brain metastasis and concurrent uncontrolled hypertension, symptomatic congestive heart failure, unstable angina pectoris, significant cardiac arrhythmia, or severe psychiatric illness were not eligible. Patients with HER2 positive (IHC 3+ or FISH/SISH +) who are potentially candidates for trastuzumab treatment were excluded. All patients provided a written informed consent according to the guidelines provided by the institutional review board.

### Treatment plan

Pazopanib was administered orally at a fixed dose of 800 mg once a day for 21 days continuously. Patients were allowed to have pazopanib as a single agent when the combination therapy was discontinued because of capecitabine- or oxaliplatin-related toxicities in the absence of disease progression. Capecitabine (850 mg/m^2^) was administered twice daily on days 1–14 and oxaliplatin (130 mg/m^2^) was administered intravenously for two hours on day one of each 21-day cycle. Patients were treated with a maximum of eight cycles of CapeOx combined with pazopanib. When patients completed planned chemotherapy or stopped chemotherapy due to other causes without disease progression, they were allowed to continue capecitabine and pazopanib until disease progression or unacceptable toxicity occurred. After 8 cycles of CapeOx plus pazopanib, whether treatment was discontinued or continued was based on investigator preference.

The primary goal of this single arm, phase II study was to evaluate the objective response (complete response plus partial response) rate (RR) in patients with gastric adenocarcinoma treated with pazopanib combined with CapeOx. The secondary end-points were progression free survival (PFS), overall survival (OS), and toxicity of the regimen.

Imaging studies for disease measurement were conducted after every two cycles of treatment for assessment of the response. The patients with a complete or partial response required a confirmatory response evaluation at least four weeks later. Patients without a confirmatory evaluation were not regarded as responders. We evaluated a response after 8 cycles for enrolled patients. Thus, overall response of this study means best response during overall treatment period. Relative dose intensity (RDI) was calculated as the delivered dose intensity divided by planned dose intensity for each drug administered. Response definitions were according to Response Evaluation Criteria in Solid Tumors (RECIST) 1.1.

According to Simon's two-stage optimal design, a sample size of 60 patients was needed to accept the hypothesis that the true RR is greater than 65% with 80% power and to reject the hypothesis that the RR is less than 50% with a 1-sided alpha of 10%. At the first stage, if there were fewer than 12 out of 23 patients, the study would terminate by rejecting the study therapy. Although the target number of patients was 60, we planned to recruit 10% more than the target number of patients considering dropout. Kaplan-Meier estimates were used for PFS and OS. Accounting for the two-stage design, the overall response rate was estimated by the uniformly minimum variance unbiased estimator [43] and its confidence interval was obtained by Jennison and Turnbull [44]. Since the final sample size is different from the planned 60, we calculated a *p*-value accounting for the two-stage design to make a decision on acceptance/rejection of the study therapy [45].
